# Adiabatic photo-steering theory in topological insulators

**DOI:** 10.1088/1468-6996/15/6/064403

**Published:** 2014-12-09

**Authors:** Jun-ichi Inoue

**Affiliations:** National Institute for Materials Science, Namiki 1-1, Tsukuba, 305-0044 Japan

**Keywords:** topological insulators, optics, external control

## Abstract

Feasible external control of material properties is a crucial issue in condensed matter physics. A new approach to achieving this aim, named adiabatic photo-steering, is reviewed. The core principle of this scheme is that several material constants are effectively turned into externally tunable variables by irradiation of monochromatic laser light. Two-dimensional topological insulators are selected as the optimal systems that exhibit a prominent change in their properties following the application of this method. Two specific examples of photo-steered quantum phenomena, which reflect topological aspects of the electronic systems at hand, are presented. One is the integer quantum Hall effect described by the Haldane model, and the other is the quantum spin Hall effect described by the Kane–Mele model. The topological quantities associated with these phenomena are the conventional Chern number and spin Chern number, respectively. A recent interesting idea, time-reversal symmetry breaking via a temporary periodic external stimulation, is also discussed.

## 1. Introduction

One of the issues attracting considerable interest in condensed matter physics is the feasible control of material properties. This theoretical concept can be directly linked to application, in areas such as materials science and device technology. Once a system is described by a model Hamiltonian that includes both intrinsic and extrinsic parameters, 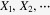, its expected physical properties are inherently characterized by these parameters and temperature. When the temperature changes, this causes a phase transition in the conventional sense. The phases involved in the transition are fully characterized by a local order parameter [1]. On the other hand, even when the temperature is fixed (usually at zero temperature), another type of transition can occur by, somehow, changing the *X*_*i*_. The phase diagram of these states is represented in a space spanned by several combinations of the given parameters. When there is a phase boundary in this parameter space, one can immediately expect a possible transfer from one state to another by external control of those parameters. A classic example can be seen in the case of superconductivity. One of the relevant parameters of this phenomenon is carrier density or equivalently, the filling factor. In the initial stages of research into this subject, the factor was varied by changing the amount of dopant used when a sample was synthesized. Recently, an alternative method for achieving this has been proposed, in which carrier injection by the application of an electric field is still possible, even after synthesis of the sample has been completed [2]. This is an example of external control of extrinsic parameters.

A possible method of controlling intrinsic parameters is to apply a temporally periodic stimulation, which will be subsequently termed ac driving, to the electron system in question. One such external stimulation is brought about by irradiation of monochromatic laser light. Leaving exemplification of efficacy of this method to section 2, here we state the primary advantage of the use of electromagnetic fields in ac driving, namely feasibility. This benefit comes from recent progress in the development of laser instruments: compactness, ease-of-use, high-tunability, and variety in choice of wavelength and output power. Although a well-known application of laser light in that research field is the control of molecular states and chemical reactions [3], it has also been applied to solid state materials. One such application is electron transport in a semiconductor superlattice driven by a laser field with a linear polarization parallel to the stacked direction. A marked effect produced by this laser irradiation is the reduction of the electron hopping amplitude between neighboring quantum wells [4–6]. Under appropriate conditions, this hopping amplitude can even vanish, resulting in electrons in each quantum well being localized. This phenomenon is termed dynamic localization, the essence of which is that the electron mass is effectively enhanced in the course of the shaking back and forth of an electron in multiple wells. A similar problem has also been examined in the case of Tomonaga–Luttinger liquid in quantum wire [7]. Hereafter, the method that enhances electron mass by irradiation of laser light is referred to as an adiabatic photo-steering. Although the idea of the method is interesting, the effect provided by adiabatic photo-steering seems to be ineffective in giving rise to a change in novel material properties, e.g., superconductivity and magnetism. This is because such quantum effects become prominent when electron correlations are strong.

However, an advent of topological insulators [8, 9] has changed the circumstances. These materials are ideal test-beds for adiabatic photo-steering since the material properties due to their topological aspects are fully described within a tight-binding model of free electrons on a lattice.

**Figure 1.**
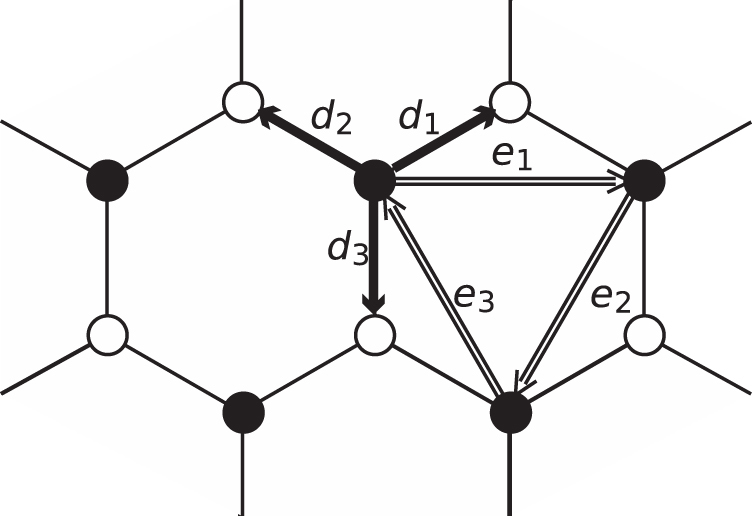
Honeycomb lattice and lattice vectors used in section 2.

A pioneering work on topological insulators was published by Haldane in 1988 [10], although it was not named as such. Here, in order partly to introduce symbols used later in this article, let us recap the results of this work [10]. The most noticeable finding is the exemplification that Landau level splitting is not necessary for the integer quantum Hall effect to occur. In a model constructed to show this fact, electrons reside on a two-dimensional honeycomb lattice comprising two triangular sublattices denoted as *A* and *B*, each of which is depicted by opened and closed dots, respectively (figure 1). An electron can hop to the nearest and second nearest neighbor sites with real amplitude, *t*_1_, and complex amplitude, 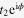, (both *t*_2_ and *ϕ* are real), respectively. The electron potentials experienced by an electron on the two sites are assumed to be different, 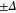, and the Hamiltonian of this system is given by

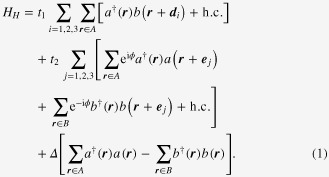


We have associated the electron creation (annihilation) operators, 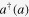 and 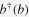, with sites *A* and *B*, respectively. The vectors, 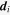 (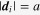: lattice constant) and 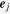


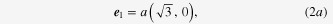


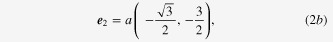


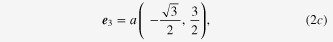
connect the nearest and next-nearest neighbors, respectively (see figure 1). The lattice constant, *a*, is set hereafter to unity, unless it is necessary. The staggered potential, 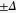, and the phase, *ϕ*, are each responsible for breaking the inversion and time-reversal symmetries. A periodic boundary condition imposed on causes the lattice to bear translational invariance. Blochʼs theorem is then applied and the electron wavefunction is characterized by a wavenumber, 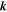. Moving in the Fourier space and expanding the Hamiltonian in the vicinity of two Dirac points

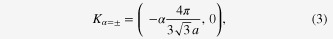
we have

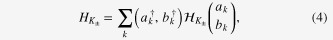


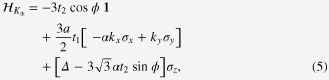
where 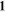 is a 2 × 2 unit matrix, 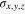 are the Pauli matrices, and the wavenumbers, 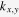, are measured relative to the 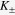 points.

**Figure 2.**
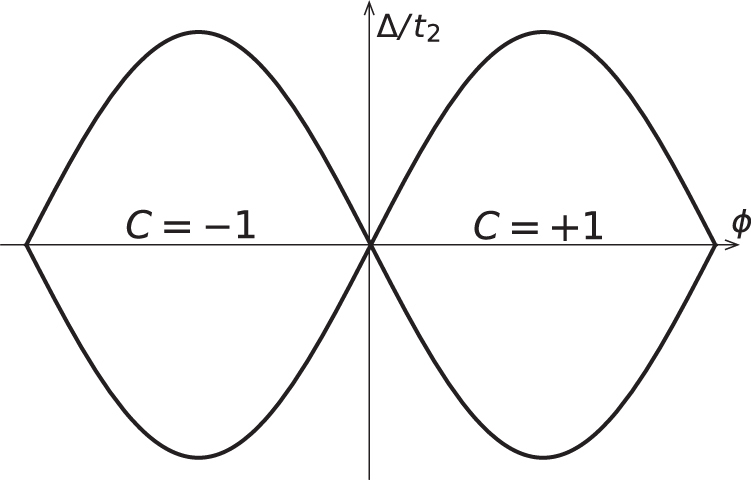
Chern number in the Haldane model. The Chern number is zero outside the curves, while the right (left) region enclosed by the two curves has 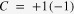.

The system is at half-filling, and is thus a band insulator, whose energy bands consist of a single valence band and a single conduction band. Let us denote the cell periodic part of the Bloch function for the lower band as 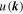, and then we define

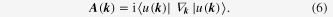
This quantity is known to mimic a vector potential in a Brillouin zone. Accordingly, it is quite natural to explore the corresponding magnetic field and magnetic flux in the Brillouin zone. Indeed

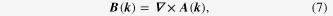


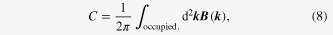
serve as those quantities, respectively [11]. The second equation, *C*, is the Chern number for the lower band. In the definition of *C*, the integral should be performed on a two-dimensional torus, 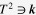. Note that, in the general context, only occupied states contribute to the integral with respect to 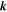. When a band in question is fully occupied, or the band is below the Fermi level (as in the present insulator case), the Chern number is proven to have an integer value. This integer is found to be associated with the Hall conductance in the integer quantum Hall state [12, 13]. On the other hand, the *C* associated with an energy band crossing the Fermi level, like a conduction band in metal, takes a general real number. The explicit calculation of *C* for the valence band of the Haldane model, equation (1), gives

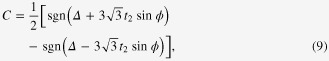
where *C* = 0 and ±1, depending on parameters, as shown in figure 2 (see the appendix for a derivation of these values).

Here, for later convenience, mention of the general properties that 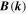 and *C* inherit from the symmetries of a given system is in order. Time-reversal symmetry leads to 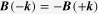, while spatial-inversion symmetry provides 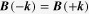 [11]. Then, in time-reversal symmetric systems, the Chern number identically vanishes, while 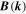 does not. Thus it is necessary to break time-reversal symmetry for non-zero Chern number [11, 14]. In contrast, when a system possesses both symmetries, 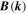 is also identically zero.

Those who observe a phase diagram such as that given in figure 2 can note that it is possible to move over two states, i.e., with *C* = 0 (conventional insulating state; outside the curves) and with 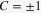 (topological insulating state; inside the curves), by externally changing a parameter, e.g., *t*_2_ [15]. Figuratively speaking, the phase diagram is a kind of a pictorial map indicating the direction that we should steer for. Since one of the parameters appearing in the phase diagram, *t*_2_, is linked to electron mass, adiabatic photo-steering can offer an ideal vehicle for exploration in this system. This is a central subject of this article, which is described in detail in section 2.1.

The aforementioned problem has no spin degrees of freedom. Since spin physics is also an important branch in condensed matter physics, adiabatic photo-steering would become more valuable if it is applicable to the control of material properties stemming from electron spins. For this purpose, it is practical to assume a non-magnetic material, since magnetic orders are dominated by strong electron–electron interactions. However, a non-magnetic material possesses time-reversal symmetry, and thus, we must begin by establishing a topological quantity that supports systems with this symmetry. The spin counterpart of the integer quantum Hall effect is known to be a quantized spin Hall effect in time-reversal symmetric systems, and a topological number that characterizes this phenomenon has been devised, termed a spin Chern number [16]. This useful quantity inherits the identical spirit with the conventional Chern number from the definition (see section 2.2), and thus adiabatic photo-steering is expected to be effective for manipulation of the quantized spin Hall current. This is another main topic of this article, which is addressed in section 2.2.

As introduced above, the role of laser light in adiabatic photo-steering is limited to electron mass enhancement. Another aspect of this approach that should be noted is that the symmetries of a bare system remain unchanged during the steering process, as seen in section 2. In contrast to this, several advanced proposals concerning further interplay between laser fields and the topological nature of materials, e.g., symmetry breaking and conversion of electron–electron interaction by an ac driving field, exist. These up-to-date topics are briefly introduced in section 3.

The last section, section 4, is devoted to addressing a rather general question: a relationship between ac driving and time-reversal symmetry breaking. The importance of a symmetry that relates both space and time is emphasized.

## 2. Adiabatic photo-steering theory in topological insulators

We demonstrate efficacy of adiabatic photo-steering in charge and spin transport phenomena using models of two-dimensional topological insulators. The required conditions for verification of the approach are clarified. A connection with a related subject, photon-assisted tunneling, is examined.

### 2.1. Photo-steering in charge transport

We implement the adiabatic photo-steering scheme in the Haldane model and manipulate the quantized Hall conductivity using a monochromatic laser light. The essence of this approach lies in the fact that the application of a circularly polarized laser field with amplitude, *E*_*T*_, and frequency, *ω*, turns the electron hopping integrals, *t*_1_ and *t*_2_, into externally tunable variables, 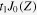 and 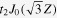, respectively, where 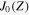 is the zero-order Bessel function and 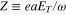 with electron charge, 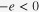. Then, through varying *Z*, or *E*_*T*_, we can tune *t*_2_ and migrate vertically in the phase diagram in figure 2, indicating a change in the Chern number of the system [17, 18]. Below, we provide a firm ground for this claim.

We add a light and matter interaction term, *H*_int_(*t*), to the Haldane model, equation (1). Within an electric dipole approximation, the interaction is represented as

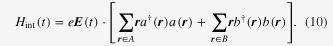
We assume that the monochromatic electric field is circularly polarized and choose the wavevector of the laser field to be normal to the lattice plane. The polarization is essential for renormalization of those hopping integrals because the applied field should uniformly disturb the electronic system. Effects by electric field with other polarizations, e.g., linear polarization, on a related honeycomb system are discussed in [19]. The driving laser field with a fixed *ω* can be expressed as

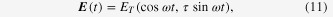
where 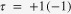 corresponds to left (right) circular polarization. Now, let the amplitude, *E*_*T*_, have sufficiently weak time-dependence to justify the use of the adiabatic approximation. Then, the sinusoidally time-dependent problem with 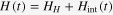, satisfying 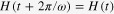, conveniently maps onto a time-independent eigenvalue problem by virtue of the Floquet technique [20, 21].

For the time-dependent Schrödinger equation with a temporally periodic Hamiltonian, 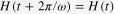


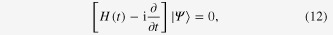
the Floquet theory assumes that the solution of this equation has the form

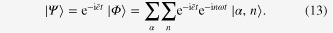
The basis used in the expansion in the second line is defined as 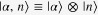, which is the composite of the appropriate electronic, 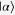, and photonic, 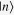, bases. The substitution of 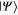 into the Schrödinger equation yields a renewed eigenvalue problem

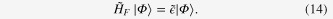
The eigenvalue, 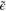, is the quasi-energy in the language of the framework.

A straightforward application of the theory to the current two-band problem (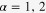) shows that the Floquet Hamiltonian, 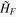, consists of 2 × 2 block submatrices 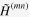, i.e.,

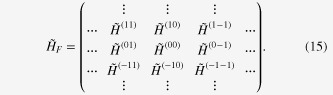
The entries for each block are defined as

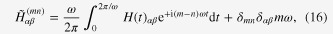
whose explicit forms are

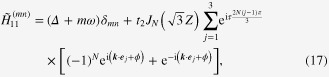


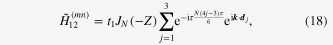


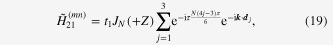


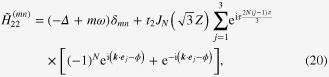
with *J*_*N*_ being the Bessel function of the 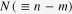
th order. During this calculation, we have performed a time-dependent gauge transformation from the length gauge to the velocity gauge [17]. This Hamiltonian operates on wavefunctions defined in the space compositely spanned by the electronic degrees of freedom and photons with energy *ω*. The diagonal block of the Hamiltonian, 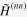, is the *n*-photon sector, i.e., the subspace with *n* photons.

When the photon energy *ω* is bounded above from the energy gap of the bare electronic system and bounded below from the hopping integrals (ideally 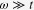), the mixture of the neighboring photon sectors is negligible. At this time, whole electron dynamics can be captured solely within the zero-photon sector. Indeed, it is observed that the quasi-energy bands given by 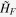 are reproduced by copying the energy bands given by the 2 × 2 matrix, 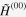. Expanding 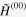 in the vicinity of the 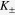 points, we arrive at the effective Hamiltonian

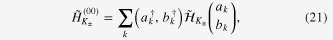


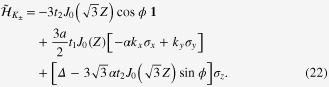
It is easy to find that 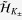 is identical in form to equation (5), apart from the replacement of the hopping integrals, *t*_1_ and *t*_2_, with 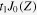 and 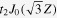, respectively. Equation (22) is the effective Hamiltonian under the influence of circularly polarized light.

**Figure 3.**
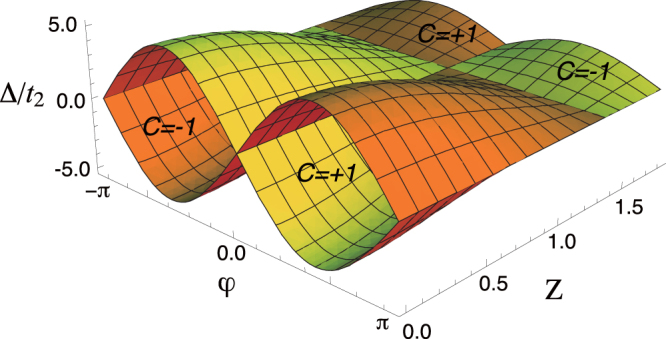
Plot of equation (23). The region inside the curved-surface has a non-zero Chern number ±1, otherwise *C* = 0. Reproduced from J-I Inoue and A Tanaka 2010 *Phys. Rev. Lett.* **105** 017401. Copyright 2010 by the American Physical Society.

The Chern number for the valence band of this renewed Hamiltonian is straightforwardly obtained as

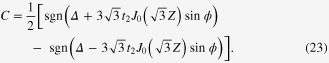
The distribution of *C* in the *ϕ*-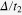-*Z* space represents the phase diagram of conventional and topological insulators (see, figure 3). The values that the Chern number can adopt are ±1 and 0, as in the model without the laser field. Although the given material parameters in the formula, *Δ* and *ϕ*, remain to be fixed, the incorporation of the new externally tunable entity, *Z*, enriches the physics. The figure of *C*, figure 3, is now manipulable by tuning *Z* that is proportional to the driving laser amplitude. One can clearly see that, in the course of the adiabatic sweep of *Z*, the Chern number changes value between 0 and 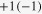. In other words, photo-induced transition occurs between the normal and Chern insulating states. This result, in turn, indicates that, in this broken time-reversal symmetric system, the circularly polarized light mimics a static magnetic field in the integer quantum Hall system [22]. In summary, we have illustrated that adiabatic photo-steering method is an effective approach to controlling charge transport, or to be specific, the integer quantized Hall current through tuning of the hopping parameters in the electronic system.

### 2.2. Photo-steering in spin transport

In section 2.1, we have shown that the quantized Hall current can be controlled using the laser field. The model used to demonstrate this is electron-spin free. Thus, a natural extension is to take the spin degrees of freedom into account. Our current scope is restricted to free-electron systems, so that we assume a non-magnetic lattice system. This in turn forces us to employ a model with time-reversal symmetry as well as translational symmetry. Therefore, we can not assert the effectiveness of adiabatic photo-steering in this system, since we can no longer rely on the conventional Chern number in the original form: the presence of time-reversal symmetry causes the Chern number to identically vanish, as emphasized in section 1.

The discussion below is divided into two cases based on spin-rotational symmetry, i.e., whether *S*_*z*_ is conserved or not (*z*-axis is taken as a spin-rotation axis). In the following, we use a minimum model Hamiltonian for an insulator to meet the purpose, which can be represented by a 4 × 4 matrix with a given wavenumber.

#### 2.2.1. The case in the presence of spin-rotational symmetry

In a topological classification of systems with time-reversal symmetry and maintaining spin-rotational symmetry, the use of the Chern number encountered in section 2.1 [17] is found to be still effective, as opposed to the aforementioned notice. This is due to the fact that the system as a whole can be regarded as consisting of two copies of spinless fermion subsystems with broken time-reversal symmetry, each corresponding to the up- and down-spin sectors represented by a 2 × 2 matrix. If the energy bands in each sector are separated by a finite energy gap, one can define a Chern number, as 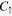 and 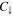, for each lower energy band in an identical manner as that of the spinless case. The sum of these two

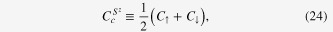
describes the net charge Hall response of the whole system. This quantity should vanish owing to the time-reversal symmetry maintained by the entire system. However, the difference

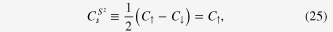
characterizing the systemʼs quantized spin Hall conductivity, need not. It is obvious that this 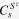 can be tunable by adiabatic photo-steering in a similar fashion as the *C* of the Haldane model, and thus the quantized spin Hall current is externally manipulable. In short, even in the time-reversal symmetric case, as long as *S*_*z*_ is conserved, the problem falls into the class studied in section 2.1, and the quantized spin Hall response can simply be inferred from the conventional Chern numbers of the individual energy bands.

#### 2.2.2. The case in the absence of spin-rotational symmetry

The situation becomes considerably complex, when the *S*_*z*_ of the system is no longer conserved. Now, the separation of the entire band structure into two independent sectors is not possible as a result of the restriction given by Kramers’ theorem, which dictates that the two subbands within the valence or conduction band become each otherʼs Kramers’ partner, and cross at time-reversal invariant wavevectors satisfying 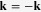, modulo a reciprocal lattice vector [23]. This interrelation prevents us from defining individual *C*_*i*_ʼs for each energy band with index 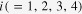. The only well-defined topological invariant is the net valence (conduction) band Chern number, i.e. 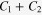 (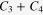) in the present four-band model, but this vanishes trivially [14]. Thus, this forces us to turn to an alternative for the topological classification of the system.

In order to define a topological invariant which is as close as possible in spirit to the Chern number classification of the *S*_*z*_-preserving case, we choose to follow the procedure proposed by Prodan [16], and concentrate on a newly introduced topological quantity, the spin Chern number. We begin by introducing an operator, 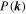, that projects elements of the original Hilbert space comprising all four energy bands, 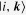, into a reduced space spanned solely by the valence band states with band indexes *i* = 1, 2

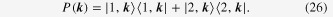
With the use of this 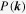, we define a projected electron spin operator

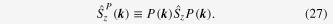
This 2 × 2 matrix operator has a positive and a negative eigenvalue. The corresponding eigenvalue problem can thus be denoted as

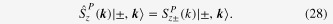
In this way, we are able to construct two projected spin spectral bands, 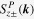. As long as these two spectral bands maintain a full gap between themselves, we can define a quantity, 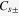, for each spectral band in a similar manner to equation (8), with

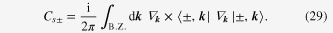
The spin Chern number of the system, which is equivalent to the *Z*_2_ topological number [24], is then constructed as [16]

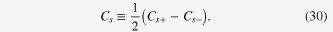
When *C*_*s*_ is non-zero (zero), the electron system is in a quantum spin Hall (trivial) insulating state. Thus, given a Hamiltonian of the electron system, one can conveniently draw a phase diagram discriminating topological insulators from their counterparts by computing *C*_*s*_ as a function of the variable parameters.

Having introduced a topological number that supports a spinfull time-reversal symmetric system, we apply it to a specific model to demonstrate the effectiveness of adiabatic photo-steering in spin transport [25]. A two-dimensional spinfull model 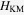 used here is that proposed by Kane and Mele (KM) [24], which is a generic description of the quantum spin Hall state. This spinfull fermion 4 × 4 matrix model is defined on a honeycomb lattice and is closely related to the tight binding model applicable to graphene and boron-nitride (BN) sheets. The model incorporates the effect of the spin–orbit (SO) interaction, of both Dresselhaus and Rashba types, and possesses time-reversal symmetry. The original form of the model was given in the real space coordinate system identical to that of the Haldane model (figure 1):

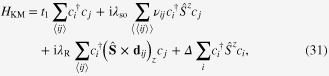
where 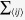 (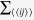) indicates the first (second) neighbor summation, and operators 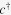 and *c* are two-component spinors. A unit vector 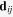 connects *i* and *j* sites, and 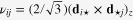, where ⋆ denotes a relay-site in electrons traversing from *i* to *j* sites.

Moving back to Fourier space, we denote the state vector by 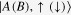, whereby an electron with up (down) spin resides on the 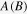 sublattice. Taking the states 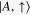, 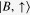, 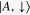 and 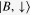 as the basis set arranged in this order, the matrix, 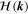, representing the KM model in Fourier space, is given as [26]

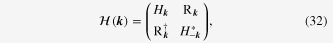
where

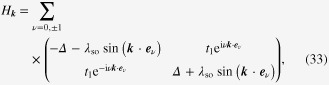


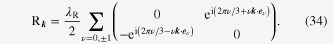
The three vectors, 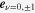, are re-indexed as 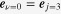, 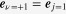, and 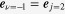 for convenience. The 2 × 2 submatrix, 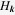, entering the diagonal block of 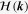 and 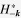 describe electrons with the up and down spins, respectively, both of which contain the kinetic energy term represented by *t*_1_, a Dresselhaus-type SO interaction, 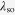, and a staggered on-site electron potential, *Δ*. The last term, *Δ*, breaks the spatial-inversion symmetry. Meanwhile, the off-diagonal entries in 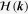 contain the Rashba SO interaction, 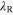, that mixes the up and down spin sectors, breaking the spin-rotational symmetry.

**Figure 4.**
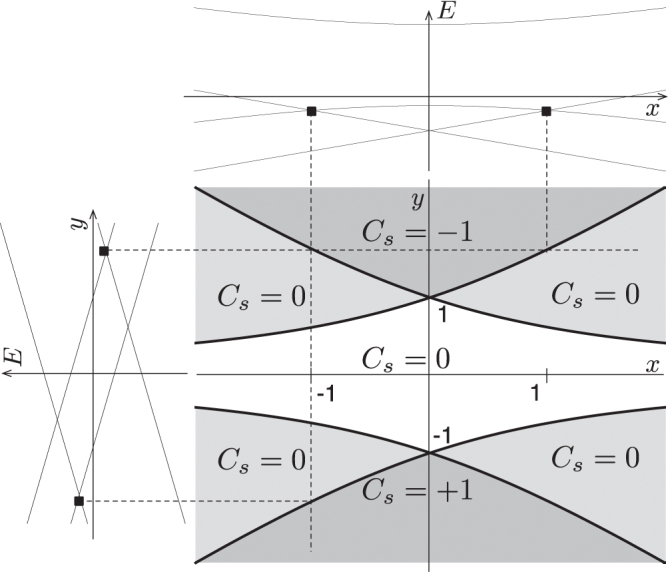
The spin Chern number distribution in the *x*–*y* plane with 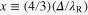, 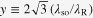. The top and side figures show the band structure of the model. Reproduced from J-I Inoue and A Tanaka 2012 *Phys. Rev.* B **85** 125425. Copyright 2012 by the American Physical Society.

A phase diagram of the spin Chern number, *C*_*s*_, for this bare system is shown in figure 4, with phase boundary given by

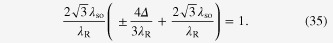
This equation is obtained as follows: from careful numerical calculation of *C*_*s*_ for the bare KM model, we find that, in an appropriate parameter space, the points at which *C*_*s*_ changes its value accompany a crossing of the second and third energy bands (see figure 4). This observation indicates that the phase boundaries of *C*_*s*_ are fixed by the energy gap closing condition, which is found to be analytically represented by equation (35).

We are now in a position to implement adiabatic photo-steering in this electron system by adding an light–matter interaction term [25]. This laser field injected from the normal direction is assumed to have an identical form to that used previously, 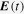, in equation (11). As seen in section 2.1, the effect of this laser field on adiabatic photo-steering amounts to the renormalization of the hopping amplitude which appears in the tight-binding description. In more general usage than previously, a hopping integral, *t*_*n*_, associated with the hopping process involving the two *n*th nearest neighbor sites, undergoes the modification 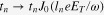, where *l*_*n*_ is the length of the straight line [27, 28] connecting the two sites. This implies that, by slowly sweeping *E*_*T*_, one can turn these material constants into externally tunable variables. This constitutes the basic idea behind adiabatic photo-steering technique, already used in section 2.1. As a specialty of the present case, we would like to emphasize that both SO interactions in the KM model, 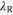 and 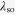, are also renormalized in a similar way

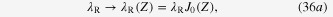


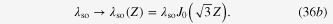
Meanwhile, the *Δ* term is left unchanged, since it is associated with an on-site potential.

One might wonder about the origin of the difference between 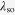 and *Δ* regarding the renormalization, since these two terms commonly appear in the diagonal part of 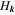. To clarify the difference, we incorporate a more physically motivated picture of the renormalization induced by the laser field, although the actual derivation of the above dependence is straightforward once the aforementioned prescription in [17] and [18] is applied. We begin by recalling that the usual minimal coupling, 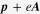, induced by light–matter interaction, where 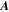 is the vector potential, is conveniently recast in the tight-binding language into a Peierls phase factor, 
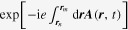
, which is associated with the hopping term involving the two sites *n* and *m*. In general, the SO interaction generates terms which include the momentum operator, 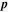, and thus the interaction is also influenced by the external field. For the case of the KM model, the Dresselhaus and Rashba SO terms take the form of second nearest neighbor and nearest neighbor hopping, respectively [24]. Hence, it is straightforward to find that, along with the kinetic terms, the coupling constants for the SO interactions are renormalized as equations (36*a*) and (36*b*).

The spin Chern number now depends, through the changes in the various coupling constants, on the newly introduced parameter, *Z*. This, in turn, will result in a shift of the phase boundaries. The latter can be read off from equation (35) upon substituting the variables with renormalized quantities. Here, in order to obtain phase boundaries which form closed surfaces (in accordance with [24]), we slightly rewrite equation (35) into another equivalent form, and obtain the explicit equations of the phase boundary surfaces as

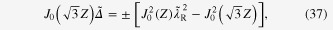
with the dimensionless variables defined as

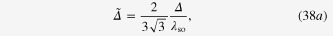


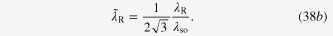



**Figure 5.**
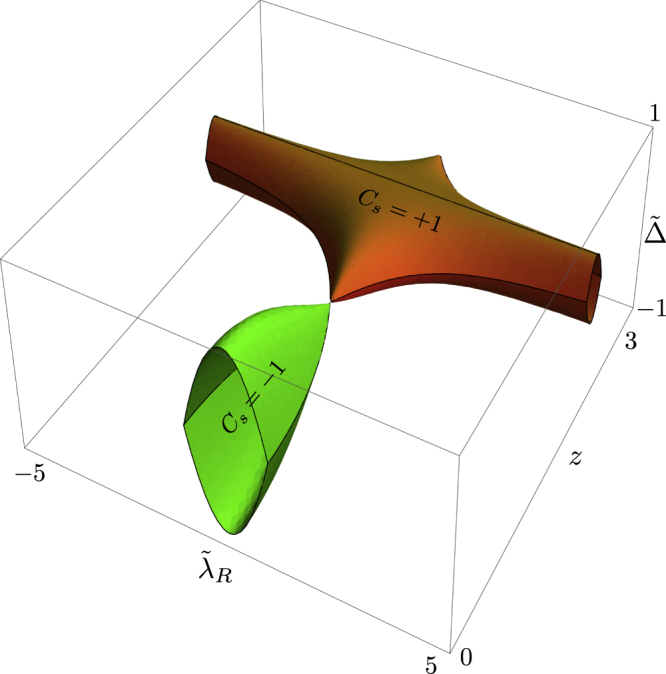
Plot of equation (37). Reproduced from J-I Inoue and A Tanaka 2012 *Phys. Rev.* B **85** 125425. Copyright 2012 by the American Physical Society.

The phase diagram which follows from equation (37) is depicted in figure 5. The case where 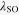 is positive is shown here, and the diagram for the negative 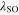 case is obtained by simply reversing the sign of the spin Chern number. First, this result, characterized by the spin Chern number, is consistent with previous work using *Z*_2_ as a topological index [24]: the section with *Z* = 0 in figure 5 is equivalent to figure 1 of [24]. As a supplementary point, one can see in *Z* = 0 plane that an area of 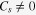 contains a line segment with 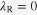. This indicates that the quantized spin Hall states with spin-rotational symmetry are continuously connected with those states without the symmetry (or 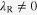). However, this fact does not imply that the use of 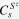 alone is sufficient for the topological classification of systems even without spin-rotational symmetry, although 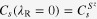. The topological equivalence between the two classes of 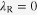 and 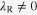 is not unveiled until the spin Chern number *C*_*s*_ has been applied, since 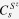 is not defined at all in the system with broken spin-rotational symmetry. On the other hand, there are states with *C*_*s*_ = 0 that are no longer continuously connected with the spin-rotational symmetric system. Thus, a change in the Rashba SO coupling induces a transition between the states with *C*_*s*_ = 0 and with 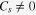.

Then, we turn our attention to adiabatic photo-steering in the quantized spin Hall current. When the system resides within the knob-like region, it is in the quantum spin Hall insulating state with 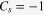, while in the bar-like region, *C*_*s*_ = 1. The two regimes cross at the the point 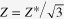, where 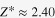 is the first zero point of the Bessel function, 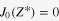. The remaining region, exterior to these two structures, is the trivial *C*_*s*_ = 0 phase. It is clear from the figure that a topological phase transition is induced upon sweeping of the laser amplitude. A system initially with 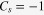 in the bare state (*Z* = 0) can be steered into a conventional insulating phase with a slow increase of the laser amplitude, and returns to the spin Hall insulating state when the external field is reduced. Furthermore, it would also appear that a transition from *C*_*s*_ = 0 to 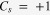 is possible, by steering a system initially lying outside the knob-like region into the bar-like region. Realizing such a steering trajectory implies that the system starts from within the profile of the bar-like structure projected onto the *Z* = 0 plane, that is, 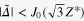. Fulfilling this condition, while at the same time keeping the energy gap, *Δ*, sufficiently large, requires a rather large 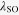. This requires in turn, however, that 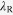 must also be large, since otherwise the bare system cannot start from outside the knob-like structure. We have confirmed numerically that certain sets of parameters can be found which indeed steer the system from 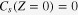 into 
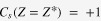
. For such parameter values, however, mainly due to the large value of 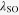, we find that the band structure of the bare system no longer exhibits a full energy gap. The system is thus expected to be semimetallic due to over-lapping of the valence and conduction bands. Even in this case, since both the energy gap and the projected spin spectral gap, still open at each 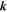, the procedure functions and the spin Chern numbers are well-defined.

### 2.3. A word on the conditions for adiabatic photo-steering

In the problem at hand, there are two independent sources of time-dependence which affect 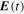: the sinusoidal behavior with the characteristic frequency, *ω*, and the temporal dependence associated with the sweeping of the laser amplitude 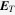. Assuming that the latter takes place on a much longer timescale than the former, one can adiabatically decouple the two: for the fast timescale of 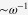, a snapshot value of 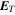 can be used. It is under this condition that the system effectively recovers time periodicity and thus benefits from the Floquet theorem. In the Floquet framework, the rapid component of the time dependence, in the present case coming from the sinusoidal oscillation, is averaged out over a period. Carrying out this averaging procedure, which involves a temporal integration, results in the Bessel function dependence mentioned above [17, 18].

After fulfilling the requirement clarified above, the parameters that characterize the adiabatic photo-steering protocol need to further satisfy the following conditions. First of all, the quantum processes incorporated in the course of the laser irradiation should involve only virtual excitations. A sufficient condition to meet this requirement is that the applied photon energy should be less than the energy gap of the original system. This condition could be relaxed depending on a specific nature of a system in question. The essence is that an electronʼs motion is confined to the valence electron bands, often referred to as the adiabatic condition on the Bloch electron [29]. Then the Fermi level stays within the energy gap during the photo-steering process, which guarantees integrability of Chern number.

A second requirement is related to the fact that our effective Hamiltonian is obtained within the zero-photon sector. This derivation is verified only when the hybridization among different photon sectors is sufficiently small. For this condition, the energy band width be smaller than the photon energy.

**Figure 6.**
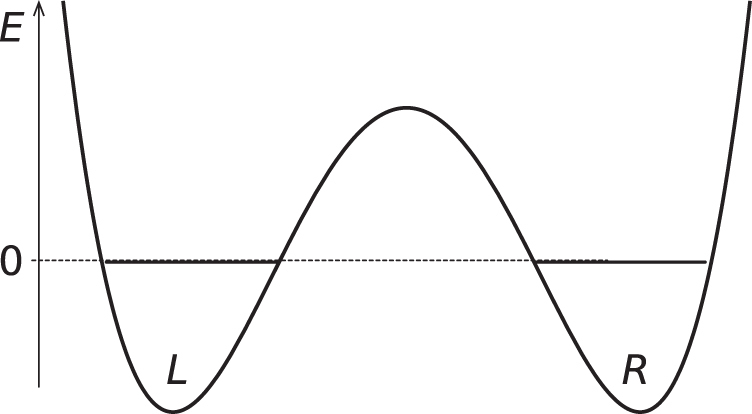
Schematics of the double well potential under consideration.

Regarding the latter condition, we would like to make supplementary comments in order to avoid possible unnecessary confusion for readers familiar with the so-called high-frequency limit. This term is generically encountered in a study of photon-assisted tunneling [30]. To begin with, let us recall vital points concerning this phenomenon by using a double potential well model. Assume that an electron is confined either in the left (L) or right (R) well, as depicted in figure 6. The electron is allowed to tunnel between the two wells with an amplitude of 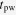. The system is described by a Hamiltonian

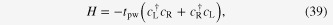
where 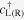 is an electron creation operator in the left (right) well. Here we add a time-dependent potential, 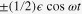, to each well, and then the total Hamiltonian becomes

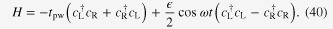
Using a localized state, 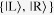, as a basis, the Hamiltonian matrix is then rewritten as

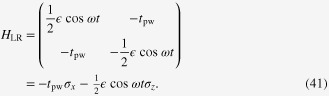
The high-frequency condition, 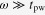, signifies that the timescale of the potential-shaking is much shorter than that of the tunneling. As a result, an electron confined in a well experiences a blurred potential energy , 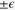, within a single tunneling event, resulting in an effective change of tunneling probability.

Next, in the basis composed of the bonding (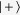) and anti-bonding states 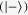, defined

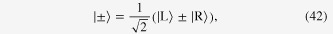
the Hamiltonian matrix becomes

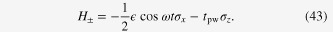
In terms of 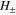, the high-frequency condition seems to indicate that sufficient energy is supplied to induce real excitation from the ‘bonding’ to the ‘anti-bonding’ states, whose energy separation is 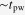. Remember that 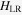, or equivalently 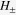, corresponds to the two unit cells problem, each of which has a single site, in the tight-binding picture of a solid.

We here connect *N* potential wells, and construct a model potential that an electron would experience in a solid. In this model, 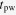 corresponds to the nearest neighbor hopping amplitude in the tight binding picture, and thus the ‘energy separation’ between the bonding and anti-bonding states in 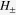 smoothly extrapolates into the band width (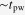) of a corresponding energy band. Thus, from the viewpoint of the tight-binding model, the high-frequency condition indicates that the band width is much smaller than the supplied photon energy, *ω*. At this point, since the unit cell has a single site, the total system falls into the class of a single band problem, and no band gap exists.

In contrast to this, the honeycomb lattice used in sections 2.1 and 2.2 has two sites in a unit cell, 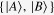, each of which has a different on-site potential, 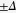. A corresponding ‘double well potential’ model is written as

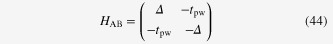


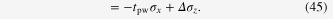
As an example of this model, 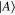 and 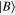
each correspond to a boron and nitrogen site in a unit cell of a BN sheet. Note that, in contrast to 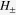, this Hamiltonian matrix describes a single unit cell containing two sites and, thus, by connecting *N* units of this item, we eventually have two electronic bands. The newly introduced energy scale, *Δ*, characterizes an energy gap between the two bands. The adiabatic condition imposed on the Bloch electron that rules out real excitation, 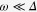, is thus independent of the high-frequency condition. Finally, we would like to emphasize another theoretical aspect that profits from this adiabatic condition, specifically, that it excludes the need for special care of relaxation processes, which, otherwise, require serious consideration.

### 2.4. Another application of adiabatic photo-steering

Adiabatic photo-steering is also effective in controlling the Berry curvature, 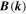, even though the corresponding Chern number is identically zero [31]. As mentioned above, a system that maintains time-reversal but breaks spatial-inversion symmetries could have finite 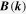. Since adiabatic photo-steering can modify an electron wavefunction by influencing electron momentum, it is therefore obvious that 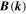 should also be controllable by this method. One of the physical quantities described by 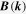 even when *C* = 0 is retained is known to be electric polarization. This classical element of electromagnetism is usually represented as an expectation value of an electron position operator. However, a shortcoming in this approach has been recognized and remedied in terms of the electric current. Intuitively, this is natural once the fact that a continuous equation relates the two is recalled. A fully-fledged theoretical framework was constructed by King-Smith and Vanderbilt [32], and Resta [33]. The 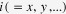
th component of the electric polarization vector *change* induced by the adiabatic photo-steering is represented, up to unimportant constants, as [31]

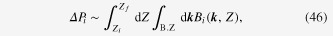
where

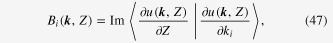
and 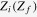 is an initial (final) value of *Z* during the steering process. One might think that this result is less significant since laser irradiation naturally induces an electric polarization in a material. However, an advantage of adiabatic photo-steering in describing electric polarization lies in the fact that it automatically surpasses the linear response regime. Indeed, the magnitude of the light and matter interaction appears as an exponential function, which signifies that the interaction is taken into account to infinite orders. These are exemplified in one- and two-dimensional systems in [31].

## 3. Further interplay between laser light and the topological nature of matter

In the previous section, we have emphasized that the influence of the external ac field in adiabatic-photo steering manifests itself as an enhancement of electron mass. During the steering process, the method preserves a symmetry endowed by the bare system. In this sense, adiabatic photo-steering enables us to work with such a *fixed* pictorial map of the topological number (as figure 2) that is inherent to the bare system.

The conditions imposed on adiabatic photo-steering are that the photon energy is less than the energy gap of the bare system and simultaneously larger than the electronic band width. Although these requisite conditions for adiabatic photo-steering verify the approach, this would obviously become more appealing if the restrictions were relaxed. In the following two subsections, we give an overview of more advanced theoretical proposals that have been independently developed, and outline potential superlative results. In particular, the last topic, addressed in section 3.2, concerns electron–electron interaction, which is out of scope of adiabatic photo-steering, and is not directly connected with the topological aspect at present. However, the effectiveness of this method holds in regards to topological materials, and we summarize it briefly.

### 3.1. Floquet topological insulator

The first example is a photo-control of topological nature of a HgTe/CdTe semiconductor quantum well by irradiation of linearly polarized light [34]. Recall that both HgTe and CdTe have the zinc-blende lattice structure. In general, materials with this structure have p-like valence and s-like conduction bands at the *Γ* point, meanwhile, HgTe has an inverted band structure with an s (p)-like valence (conduction) band. Accordingly, in a quantum well consisting of these two materials, the energy subbands generated inside the well region may inherit the band inversion, depending on their well width at the nano-scale. The essence of the topological transition in the HgTe/CdTe quantum well is the occurrence of the subband inversion, as recognized in [35]. When the thickness of the HgTe layer sandwiched by the CdTe layers is larger (smaller) than a certain critical value 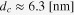, the quantum well becomes a topological (trivial) insulator. Quantization of spin Hall current was theoretically predicted as manifestation of this topologically non-trivial nature, and has been experimentally confirmed [36].

When an ac driving laser field is added to this system, the field serves as a knob that can be used to effectively tune the quantum well width [34]. Using linearly polarized light with energy *larger* than the energy gap, the relative positions of the subbands are found to be controlled on the basis of the Floquet theory. Thus, one can induce a topological phase transition in a quantum well sample with a given well width, and the advantage of this approach is obvious. Experimental realization of this phenomenon has been awaited. Further development along this line includes an extension to a scheme that incorporates two-photon resonances [37]. Recently, this kind of topological insulator, which is newly generated from a topologically trivial insulator through an interplay with the ac field, has been referred to as a Floquet topological insulator [34, 38–42], and it now forms a sub-branch in the field of topological materials.

A more striking effect of an ac driving field would be to totally redraw a phase diagram of Chern number. This inevitably breaks time-reversal symmetry by the external field. For instance, a phase diagram that is entirely occupied by an area with *C* = 0 as a intrinsic property of the original system, is re-constructed, and turns into a diagram incorporating finite areas with 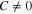. This means that the original material is transformed into a topological one in a true sense.

This idea was considered in relation to graphene in [43], and related work on graphene and graphene ribbon can be seen in [44–48]. Since a model for ideal graphene incorporates both time-reversal and spatial-inversion symmetries, this is a gapless system whose band structure forms a Dirac cone centered at the 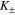 points in the Brillouin zone. Once a circularly polarized continuous laser light is normally injected, the electronic band structure obtains a finite energy gap at the 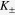 points. The magnitude of this newly induced gap is proportional to 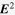, and thus the effect is recognized as a second order process. Note that this gap creation is distinct from the famous ac Stark effect [49]. If it is due to the ac Stark effect, the gap would open at a certain 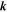 point at which a vertical transition occurs from a valence band to conduction bands induced by the applied laser field. This 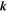 point should differ from the 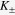 point as long as the ac field has a finite frequency.

When a finite gap opens at the 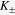 points, this gap in turn serves as an energy gap between the Landau sub-levels in the conventional quantized Hall state, and therefore, the Chern number associated with this system can have a non-zero integer value [43]. Since the bare system surely possesses time-reversal symmetry, the emergence of the finite Chern integer indicates that the symmetry has been broken by the ac field. The origin of this symmetry breaking could be ascribed to circulation of the polarization plane of the laser light^1^.

Consequently, graphene, a topologically trivial system, becomes a topological insulator once the circularly polarized light is applied. To express this differently, the role of photo-irradiation is to redraw the phase-diagram of the topological number determined by the bare system. This novel effect cannot be obtained when the theoretical framework is limited to within one-photon sector in the Floquet Hamiltonian [39].

A recent related experimental achievement is also worth mentioning. For two-dimensional gapless Dirac cones defined as surface states of a three-dimensional topological insulator, 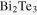, Wang *et al* reported an observation of a finite gap opening (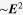) at the Dirac points following circular photo-irradiation [50]. This phenomenon is considered to lie in a context of the Floquet topological insulator^2^.

### 3.2. Control of an electron interaction by an external field: conversion between the repulsive and attractive force

We might immediately think that the control of an electron interaction by a certain external means would be quite valuable. In this regard, a curious theoretical proposal has been put forward [51]. According to theory, the magnitude of the Coulomb interaction in a strongly correlated system should change by continuous laser irradiation. In particular, this change includes conversion of the interaction from repulsion to attraction. Thus, one can reasonably expect possible photo-control of superconductivity and magnetism in strongly correlated systems.

One might notice a similarity with the interaction change between cold atoms. The mechanism of the atomic interaction change, including its qualitative aspect, relies on Feshbach resonance. The internal degrees of freedom established in atomic energy levels is relevant to the nature of the interaction between atoms. By tuning the applied magnetic field, the energy structure inside an atom is modified, resulting in a change in the interaction. This phenomenon, or equivalently a change of scattering length, was experimentally confirmed in a solidum Bose–Einstein condensate [52].

However, the idea proposed in [51] is totally distinct from the technique used in cold atom physics. The authors of the paper make full use of the equivalence between a repulsively interacting electron system at a negative temperature and an attractively interacting electron system at a positive temperature. The issue that must be overcome is how the negative temperature state can be realized. Although a negative temperature state is not permitted at a thermal equilibrium condition, the situation varies drastically under non-equilibrium conditions. The authors of this study argue that they can construct an appropriate non-equilibrium state with negative temperature by irradiation of a continuous laser light. The situation is similar to laser emission, where a negative temperature state is achieved through population inversion of a non-equilibrium state. More recently, they have further developed their theory and claim that interaction conversion is possible with a pulsed laser, instead of continuous light [53]. A principle focus of these proposals to date has been superconductivity, but this method would enable one to externally control the topological nature of materials enriched by an electron–electron interaction.

## 4. AC driving and time-reversal symmetry breaking

In this final section, we would like to introduce a rather generic problem that can be accessed through the small window of *the external control of material properties*. The question concerns symmetry breaking caused by an external ac field.

### 4.1. Generality of the problem

The problem of symmetry breaking using an external field has been studied from diverse view points in various research fields, so that topics which are mentioned here are quite limited. The research fields where the problem has been addressed, to name only a few, are classical and quantum ratchets [54–56], quantum chaos [57, 58], and artificial gauge fields in optical lattices [59].

In ratchet problems, one of the essential problems is how to generate a directional flow in a uniform system by non-directional ‘shaking’. Through extensive studies within classical physics, the importance of space-time symmetries, such as generalized parity and generalized time-reversal symmetry, has been recognized [54–56]. A similar concern is also crucial to the quantum version of this problem [60]. A quantum system whose classical correspondence shows chaotic nature is fully classified by the symmetry of a given Hamiltonian into three ensembles: orthogonal, unitary, and symplectic, using the terminology of Dyson [61]. The first and third ensembles possess time-reversal symmetry, while the second does not. A pertinent question is whether an orthogonal ensemble system driven by an ac field that varies for 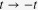 alters into a unitary ensemble system or not. According to [60], the simple addition of sinusoidal driving, 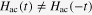, e.g., 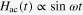, to an orthogonal ensemble system does not transform it into a unitary system. This counter-intuitive finding is recognized as a result of the space-time symmetry inherent to the system [62].

While keeping the general aspects mentioned above in mind, let us return to our problem, which shares common features with the artificial gauge generation problem in cold atom physics [63].

### 4.2. Complex effective hopping amplitude

The core principle of adiabatic photo-steering is that the effect of a driving ac field manifests itself in the renormalization of parameters for electron hopping and spin–orbit coupling, otherwise they are similar to material constants. We further note that, during the steering process, the reality of the parameters is maintained. Here, a natural question is raised, i.e., if an effective hopping parameter could possibly become a complex number as a result of ac driving [64]. If this is the case, this implies that the ac field will generate an artificial magnetic field, which would then break the time-reversal symmetry of a given electronic system.

The *necessary* condition for which an effective hopping parameter would become complex is pinned down in [65]. The ac component of the Hamiltonian, which is written in the form of temporally periodic lattice shaking, 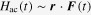, with 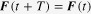, must simultaneously break the following two symmetries: (a) reflection symmetry with respect to a certain time, *τ*; 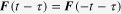 and (b) shift symmetry; 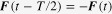. Therefore, a single sinusoidal driving never creates an effective magnetic field, although this was proposed in [66, 67], and, therefore, other approaches are required [65, 68]. Note that a model to show that the above conditions are not *sufficient* is presented in [69].

In accordance with the argument, since the ac driving field used in sections 2.1 and 2.2 processes the shift symmetry, the time-reversal symmetric properties inherent in the models must remain unchanged. Indeed, the original Haldane model in section 2.1 breaks the time-reversal symmetry because 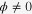, and as does the effective Hamiltonian, equation (22), obtained through the adiabatic photo-steering. The KM-model used in section 2.2, which possesses time-reversal symmetry, becomes effective Hamiltonian with the symmetry. Thus, the approximations used in constructing the adiabatic photo-steering protocol fully respect the results drawn from the general considerations.

### 4.3. Effect of the initial phase of the driving field

On a related note, one might observe that an initial phase, *α*, in a driving ac field, 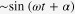, would have an influence on the effective hopping amplitude. Indeed, there are some reports that highlight a substantial effect of the initial phase [70, 71]. According to these studies, when the system is driven by a cosinusoidal field 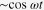, the effective hopping parameter remains real, while, when driven by a sinusoidal field, the parameter becomes complex [70]. This result appears reasonable, because 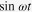 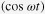 function alters (maintains) its sign under a trivial time-reversal operation, 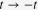, and thus the complexity (reality) of the obtained hopping parameter would be consistent with the behavior of the trigonometric functions under the time-reversal. According to the gauge principle, however, no physical effects should depend on the initial phase. In the following, to confirm this fact, we observe how *α* influences the effective hopping parameter by using, once again, the double potential well model [64].

Consider a time-dependent Hamiltonian, *H*(*t*), for an electron in a double well potential driven by external fields, *F*(*t*), such that

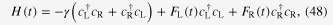
where a real parameter, *γ*, quantifies the electron hopping amplitude between the two wells. Performing a time-dependent canonical transformation, *U*(*t*)

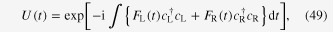
on both sides of the Schrödinger equation

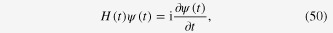
we then obtain a renewed equation for 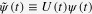


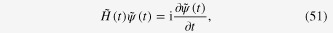
where the transformed Hamiltonian is found to be

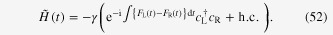
For monochromatic driving with frequency *ω* and an initial phase *α*

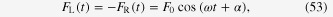
the hopping parameter accompanies a time-dependent phase

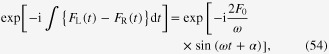
and thus, the effective hopping parameter reads

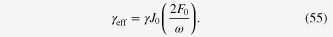
This is understood under the high-frequency condition by obeying the usual treatment of the Floquet formulation, i.e., taking the average over one temporal period (see sections 2.1 and 2.2), or taking the leading term of the well-known expansion

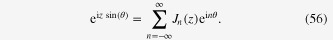
The point to be emphasized is that, in any case, the effective hopping parameter remains real and *α*-dependence does not appear.

Here, instead of *U*(*t*), one can perform a similar but slightly modified canonical transformation, 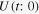, defined as

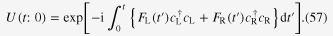
A parallel calculation then yields an effective hopping parameter, 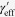, which is now found to become complex, depending on *α*:

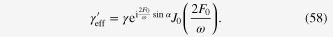
As seen in its derivation, the origin of the difference between 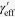 and 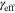 lies in the value of 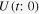 evaluated at 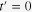. The appearance of this complex parameter would manifestly indicate time-reversal symmetry breaking caused by the ac driving field. However, it is rather strange that time-reversal symmetry breaking depends on the choice of canonical transformation, *U*(*t*) or 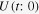.

In fact, the extra phase factor appearing in 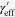 has no substantial influence on the full time-dependent solutions of the Schrödinger equation. Below, we show this by following the discussion presented in [64]. The explicit form of the Schrödinger equation with the Hamiltonian given in equation (48) is

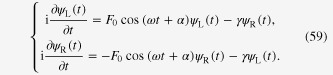
In order to find a solution, we use an ansatz

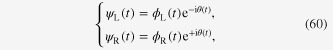
where

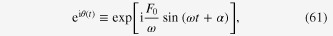
and then we have

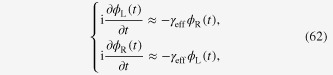
after taking the average over one period of *t*. Corresponding matrix Hamiltonian and the normalized eigenvector for the lower energy state are

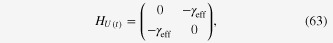
and

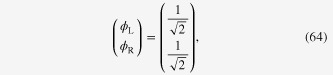
respectively.

On the other hand, we can use the following alternative solution ansatz

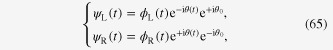
where 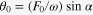 is assumed. In this case, the Hamiltonian matrix is found to be

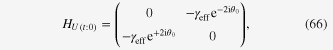
with normalized eigenvector of

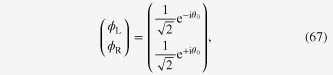
for a lower eigenenergy state. Depending on the solution ansatz used, the eigenvectors are different. However, we can immediately observe that the full time-dependent solutions, 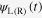, are identical in both cases. It is now obvious that use of the first or the second solution ansatz corresponds to performing the canonical transformation *U*(*t*) or 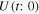, respectively. This sort of ambiguity stems from a choice of the origin of time, and one should devise a time-dependent canonical transformation scheme that is free of this problem. One such solution is presented in [72], where one- and two-dimensional electronic systems are examined to show their effectiveness. In conclusion, the extra phase factor appearing in 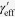 using 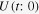 has no physical effect, and the time-reversal symmetry inherent to the undriven system is maintained during the monochromatic ac driving.

The above argument holds if and only if the high-frequency limit is verified, since the derivation relies on an assumption that the zero-photon sector effectively describes low-energy physics. Indeed, the effective hopping parameters are derived through taking an average over one period with respect to time. However, several studies exist, as introduced in section 3.1, which show that even single monochromatic laser irradiation surely breaks the time-reversal symmetry of an electron system, once one-photon sector has been surpassed. The relationship to the conditions in section 4.2, which are derived from the general discussion in terms of symmetries, is of interest. Keys to solve the problem may lie in differences between continuum or discreet and/or single- or multi-band systems.

## 5. Conclusion

We have reviewed an optical method for feasible external control of material properties, termed adiabatic photo-steering. The essence of the method lies in the fact that the influence of the applied laser field manifests itself as the electron mass enhancement, resulting in changing the electron hopping amplitude. Efficacy of this approach is demonstrated in two-dimensional models for topological insulators, through the integer Hall effect and the quantum spin Hall effect. More advanced theoretical methods, Floquet topological insulator and conversion of electron interaction, were introduced. Finally, as a general problem accessed from the central subject of this article, symmetry breaking via ac driving has also been discussed.

## Footnotes

1 Remark that a time-reversal operation on the left (right) circularly polarized light propagating along one direction provides the left (right) circularly polarized light propagating along the opposite direction, since wavevector changes sign through the time-reversal operation. This is consistent with the fact that helicity, defined as the inner product of the wavevecor and spin operator, is even against a time-reversal operation, since each is odd for the operation.

2 A finite gap at the Dirac point can be seen even in a time-reversal symmetric system, e.g., a BN sheet.

## Appendix. Calculation of a Chern number in the Haldane model

Consider a 2 × 2 Hamiltonian matrix

The eigenvalues of this Hamiltonian, , are not degenerate unless . Then, the Chern numbers associated with each band are well-defined and, for the lower band, are expressed as

The most comprehensive derivation of this result is given in [73]. Since the effective Hamiltonian of the Haldane model in the vicinity of the  points is written in the above form, the Chern number associated with a band below the finite energy gap is the sum of the two, such that

Thus, for a non-zero Chern number, it is required that the signs of the ‘masses’ between the  points must differ.

However, one might have an interest in Chern number calculation without dividing a single band into two elements. For this purpose, among several methods for the Chern number calculation [74–76], we apply a procedure presented by De Nittis and Lein to reproduce the diagram of Chern number for the Haldane model [77]. We first rewrite the model into the following convenient form, which is a linear combination of the Pauli matrix , with

where  and . In the above equation,  is a point on *T*^2^, and the energy is normalized as . From the general consideration, the projection operator onto a lower energy band is [78]

where

In terms of , the connection one-form, , and curvature two-form, , are represented as , and , respectively [79]. Since a Chern number is given as the integral of the curvature on the base manifold in question, we can expect that the Chern number is obtained solely from the projection operator  and, indeed, this is the case. Since

we now have a natural map from  to a two-sphere, , both of which are related as

For a Chern number to have a non trivial value, there must be points on *T*^2^, one of which can be mapped onto the ‘north pole’, , and another of which onto the ‘south pole’,  on *S*^2^. The poles are given by the condition , resulting in

Furthermore, if ,  is mapped to the ‘north (south) pole’, and thus  is required for a non-trivial Chern number, that is,

The Chern number itself is given by the number of times the pole is surrounded by the map:

As an example, we calculate *C* of the case where  is mapped to the north pole. In order to perform the integration, we take a path that surrounds the north pole, where

Evaluation of  in the vicinity of  expanding up to , leads to

Accordingly, the Chern number is obtained as

Similarly, the other case where  is mapped to the south pole provides us with

Then, we have completed the re-derivation of the Chern number for the Haldane model.
